# Harnessing the Power of T Cells: The Promising Hope for a Universal Influenza Vaccine

**DOI:** 10.3390/vaccines6020018

**Published:** 2018-03-26

**Authors:** E. Bridie Clemens, Carolien van de Sandt, Sook San Wong, Linda M. Wakim, Sophie A. Valkenburg

**Affiliations:** 1 Department of Microbiology and Immunology, The University of Melbourne, Peter Doherty Institute for Infection and Immunity, Melbourne, VIC 3000, Australia; bridie.clemens@unimelb.edu.au (E.B.C.); cvandesandt@unimelb.edu.au (C.v.d.S.); linda.wakim@unimelb.edu.au (L.M.W.); 2 Department of Infectious Diseases, St. Jude Children’s Research Hospital, Memphis, TN 38105, USA; Sook-San.Wong@STJUDE.ORG; 3 HKU Pasteur Research Pole, School of Public Health, University of Hong Kong, Hong Kong 999077, China

**Keywords:** T cell, influenza virus, universal vaccine

## Abstract

Next-generation vaccines that utilize T cells could potentially overcome the limitations of current influenza vaccines that rely on antibodies to provide narrow subtype-specific protection and are prone to antigenic mismatch with circulating strains. Evidence from animal models shows that T cells can provide heterosubtypic protection and are crucial for immune control of influenza virus infections. This has provided hope for the design of a universal vaccine able to prime against diverse influenza virus strains and subtypes. However, multiple hurdles exist for the realisation of a universal T cell vaccine. Overall primary concerns are: extrapolating human clinical studies, seeding durable effective T cell resident memory (Trm), population human leucocyte antigen (HLA) coverage, and the potential for T cell-mediated immune escape. Further comprehensive human clinical data is needed during natural infection to validate the protective role T cells play during infection in the absence of antibodies. Furthermore, fundamental questions still exist regarding the site, longevity and duration, quantity, and phenotype of T cells needed for optimal protection. Standardised experimental methods, and eventually simplified commercial assays, to assess peripheral influenza-specific T cell responses are needed for larger-scale clinical studies of T cells as a correlate of protection against influenza infection. The design and implementation of a T cell-inducing vaccine will require a consensus on the level of protection acceptable in the community, which may not provide sterilizing immunity but could protect the individual from severe disease, reduce the length of infection, and potentially reduce transmission in the community. Therefore, increasing the standard of care potentially offered by T cell vaccines should be considered in the context of pandemic preparedness and zoonotic infections, and in combination with improved antibody vaccine targeting methods. Current pandemic vaccine preparedness measures and ongoing clinical trials under-utilise T cell-inducing vaccines, reflecting the myriad questions that remain about how, when, where, and which T cells are needed to fight influenza virus infection. This review aims to bring together basic fundamentals of T cell biology with human clinical data, which need to be considered for the implementation of a universal vaccine against influenza that harnesses the power of T cells.

## 1. Introduction

Countless examples exist for influenza A viruses causing havoc on public health, from perpetual seasonal epidemics, worldwide pandemics, and zoonotic infections from animal reservoirs, yet our current vaccine methods do not arm us against the diversity of influenza viruses. Influenza vaccines are the most widely used vaccines in the world, with over 500 million doses used annually [1], due to seasonal epidemics and the recommendation of annual vaccination. However, the efficacy of the inactivated influenza vaccine (IIV) is moderate to poor, and is impacted by antigenic drift [2], mismatch [3,4], pandemic emergence due to reassortment [5], and egg adaptations during vaccine production [6], which can all lead to reduced protection and increased incidence of infections. The efficacy of the live attenuated influenza vaccine (LAIV)—mainly recommended for use in children—has also dropped in recent years [7], possibly due to thermal stability issues [8] or antigen competition during priming [9]. Overall, these factors have culminated in reduced public confidence in influenza vaccines [10].

Current vaccine stockpiles for avian influenza viruses H5N1 and H7N9 have reduced immunogenicity compared to seasonal influenza viruses [11,12], requiring multiple doses, the use of adjuvant, and may not match future emergent versions of these viruses [13]. The 2009 H1N1 pandemic showed that we are only able to respond after the fact, as the monovalent pandemic vaccine became available after the peak of human infections, leaving the majority of the population to “ride out the storm” and public outcry at the spectre of the pandemic severity predictions. Vaccine production methods have been significantly ramped up in the wake of the 2009 pandemic, but the timing of virus isolation, distribution, and large-scale production will encounter similar issues in future pandemics. Overall, a substantial revitalisation of the current vaccination program is needed to combat influenza viruses, overcome vaccine production limitations, and pre-arm ourselves against diverse and divergent influenza A viruses.

## 2. Basics of T Cell Responses during Infection and Vaccination

**Figure 1:**
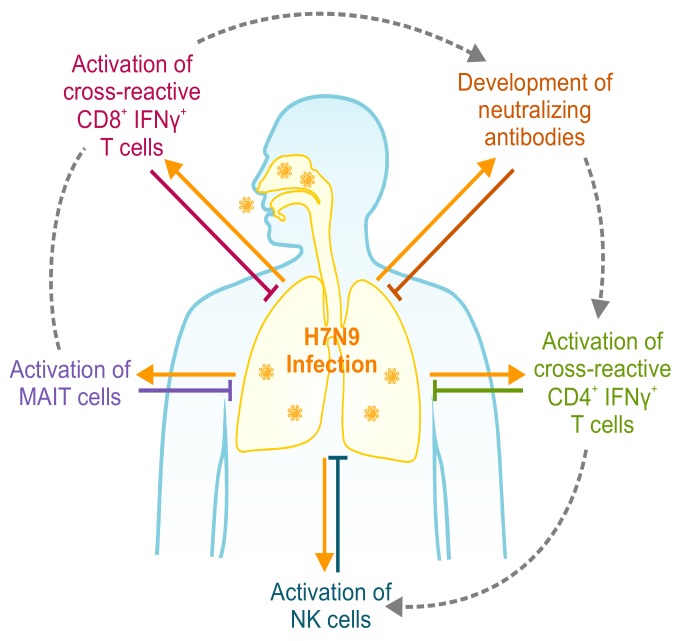
CD4 and CD8 T cells act in synergy with multiple immune arms for heterologous protection. Effective heterologous immunity against zoonotic influenza (H7N9) viruses requires synergy of multiple immune arms [30,76,78]. Without the recruitment of two or more immune arms, protective immunity is diminished, as modelled on outcomes of infection from H7N9-infected patients. Although multiple arms are likely to be activated at the same time, hospitalized patients clearly demonstrate that different arms had a more prominent role if one arm fails to respond. MAIT: mucosal associated invariant T.

**Figure 2:**
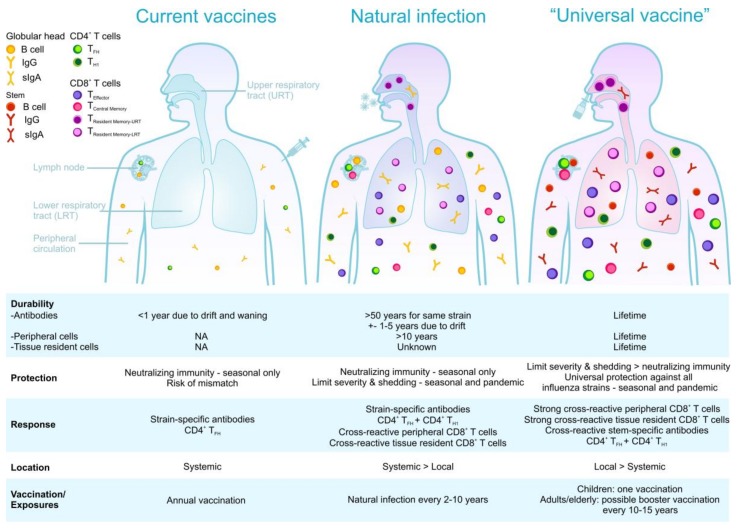
Immune responses stimulated by natural influenza virus infection, current vaccination, and the ideal scenario of a universal vaccine.

Vaccination educates our adaptive immune system—specifically T and B cells—for a faster, stronger, and more specific response upon re-encounter with the matching antigen. However, current IIVs and LAIVs are not efficient in inducing T cell immunity, potentially contributing to their limited efficacy and breadth of reactivity against diverse influenza viruses. Importantly, current inactivated influenza vaccines tend to prevent the induction of cross-reactive CD8^+^ T-cells, which would otherwise be elicited by natural influenza virus infections and are our primary protection in case of a vaccine mismatch or pandemic outbreak [14] (Figure 1 and Figure 2).

T cells express a clonal T cell receptor (TCR), which recognizes linear fragments of viral peptides that are 9–15 amino acids long, presented by the major histocompatibility complex (MHC) molecules on the surface of infected cells or antigen presenting cells. Access to the MHC presentation pathway utilises endogenously (direct presentation) or exogenously (cross-presentation) derived peptides generated by the cleavage of viral proteins by the immunoproteasome. This process has important implications for vaccine approaches that need to consider the design and delivery of antigen to these pathways for the activation of T cells. Viral entry and translation are required for access to MHC I processing machinery; however, peptide presentation can precede virus replication. The protein composition and immunodominance hierarchies of the subsequent T cell responses predominantly reflect abundant virion proteins expressed during infection and the timing of that expression [15].

Memory T cells can provide very effective heterosubtypic immunity, whereby T cells are able to recognise different and even unrelated influenza viruses due to peptide homology or the conservation of linear peptide sequences between different strains and subtypes of influenza [16,17,18,19,20,21]. Heterosubtypic T cell immunity is especially important when there is no existing antibody response, which can occur during seasonal antigenic drift, zoonotic infection, or a pandemic situation. The protective efficacy of cross-reactive CD8^+^ T cells has been demonstrated in extensive animal studies of mice [22,23,24] and ferrets [25]. For example, mice primed with H3N2 virus, boosted with H1N1, and finally challenged with a high dose of lethal H7N7 virus have no detectable virus replication [26]. Thus, unlike the majority of B cells, T cells are capable of expanding immunological protection against diverse influenza viruses.

Although antibody-mediated immunity elicited by natural infection or current vaccine strategies is capable of providing sterilizing protection, this protection is generated primarily against the hemagglutinin (HA) and neuraminidase (NA) epitopes on the virus surface. Immune escape is more common for surface antigens via modifications of glycans and amino acid substitutions at key antigenic sites than internal proteins which are constrained by functional limitations of viral fitness [27,28]. Lee et al. identified 72 T cell peptide epitopes that are cross-recognised between H5N1 and seasonal H3N2 viruses by CD4^+^ and CD8^+^ T cells, and only one was derived from the HA surface protein [17]. The majority of T cell epitopes are derived from internal proteins, which have a conservation rate of >90% between different IAV strains and subtypes, whilst HA and NA surface proteins are only 40–70% conserved between different IAV subtypes [29]. Indeed, T cells that are cross-reactive against highly pathogenic H7N9 and H5N1 avian influenza viruses can be found in the peripheral blood of unexposed healthy volunteers [17,19,20]. Furthermore, robust and expedient recruitment of such cross-reactive T cell memory correlates with improved infection outcomes, faster recovery, and survival from H7N9 infection [30].

While influenza A and B viruses share many features, there is little sequence identity between analogous proteins (range 7–37% amino acid identity), with the exception of the polymerase basic 1 (PB1) protein (58% amino acid identity) [31]. Despite this, broadly neutralizing monoclonal antibodies against highly conserved HA and NA epitopes of influenza A and B viruses have been reported (reviewed in [31,32]). In addition, a conserved CD4^+^ T cell epitope in the HA fusion peptide of influenza A and B viruses can elicit cross-reactive CD4^+^ T cells in humans, and a number of well-characterized CD4^+^ and CD8^+^ T cell epitopes in the PB1 protein show complete amino acid conservation or only slight variation amongst influenza A and B viruses [31,32]. This hints at the possibility of truly universal pan-influenza A and B virus T cell immunity that could be harnessed by vaccination. The area of research on T cell cross-reactivity beyond influenza A viruses currently remains underexplored.

T cells are represented by a family of phenotypically diverse subtypes in terms of function, location, and magnitude. Cytotoxic CD8^+^ T cells recognize viral peptide in the context of MHC class I (MHCI), which is expressed on the surface of all nucleated cells, allowing immune surveillance. CD8^+^ T cells mediate the direct lysis of virus-infected cells and produce anti-viral cytokines, effectively and directly reducing viral load and the length of infection. Meanwhile, helper CD4^+^ T cells recognize viral peptides within MHC class II (MHCII), which is expressed by professional antigen presenting cells, B cells, macrophages, dendritic cells, and other CD4^+^ T cells. Helper CD4^+^ T cells are a critical cornerstone of establishing effective immune memory against influenza virus infection, and are necessary for the establishment of CD8^+^ T cell memory responses [33] and high avidity class switched antibodies (reviewed in [34]). In addition, it has been proposed that MHCII is also expressed by type II alveolar pneumocytes during infection [35], enabling CD4^+^ T cells to target a minor population of infected cells in the lung tissue by direct cytotoxic mechanisms themselves.

T cells express a clonally diverse and highly specific TCR on their cell surface. The TCR consists of an α and β chain heterodimer, with the fine specificity of antigen recognition provided by somatic recombination and non-germ-line encoded additions to the complementarity determining regions (CDRs) of each chain. The estimated diversity of the human T cell repertoire is 2 × 10^7^ distinct TCRs [36], whilst the TCR diversity of an individual epitope-specific T cell response can be oligoclonal or composed of up to 40 different distinct TCR sequences [37,38]. Unlike antibody responses, TCRs do not undergo affinity maturation and the thymus involutes after puberty, greatly diminishing the output of naïve T cells as we age. The capacity for heterologous immunity by T cell responses is imperative for protection against the huge array of possible pathogens encountered in a lifetime, which out-numbers the TCR repertoire; therefore, an overlap in TCR specificities is a necessity. Heterologous T cell protection to different epitopes has been reported in mice between LCMV, vaccinia virus, and Pichinde virus [39]; and in humans between influenza and hepatitis C virus [40]; papilloma viruses and coronaviruses [41]; influenza and Epstein–Barr virus (EBV) [42,43]; Dengue subtypes [44]; and as discussed above, between different strains of influenza and variants of immunodominant influenza-derived epitopes [45]. 

T cells are derived from the bone marrow progenitor cells, mature and develop in the thymus, undergo positive and negative selection processes, then emigrate as non-self-reactive immature cells in the periphery for immune surveillance for cells expressing altered or non-self-antigens. Immature naïve T cells circulate between secondary lymphoid organs surveying for cognate antigen, while mature memory subsets—depending on their phenotype and priming signals—can reside in the tissue parenchyma (T cell resident memory, Trm), the lymph node (T cell central memory, Tcm) and periphery (T cell effector memory, Tem). T cell recognition of peptide-MHC in the context of further signalling (e.g., co-stimulation and inflammatory cytokines) leads to T cell activation, proliferation, and differentiation, amplifying the effective army of T cells against infection.

The number of naïve epitope-specific CD8^+^ T cells has been estimated in the mouse model for immunodominant and subdominant epitopes at 39–72 cells/mouse and 247–315 cells/mouse, respectively [38]. The efficiency of recruitment from the naïve pool of T cells to respond during acute infection is dependent on numerous factors, such as: the peptide context, MHC allele restriction, antigen presenting cell, the timing of antigen expression during the virus life cycle, and the avidity of the TCR and peptide–MHC interaction ([46], and reviewed in [47]). After TCR engagement, recruitment, and activation, naïve T cells then amplify up to 10,000-fold during the acute stages of infection. The rapid expansion and peak magnitude of virus-specific T cells coincides with drastic reduction in viral titres from days 5–9 of primary virus infection, whereas influenza-specific antibodies peak and plateau from days 14–20 post infection. Following antigen clearance at about 14 days post influenza infection, the antigen-specific T cell pool contracts, whereby the most differentiated cells undergo activation-induced cell death by apoptosis, leaving behind a stabilised memory pool [48]. The kinetics of T cell responses in human influenza infection are more variable, with some studies reporting a rapid peak in virus-specific T cells at one-week post-infection with H1N1 that subsequently contracts within 3–4 weeks after infection [22,49], while others suggest a more protracted response dynamic, peaking 3 weeks after infection with only modest decline in cell numbers by day 78 and returning to baseline by day 700 [50]. To what extent these variations in dynamics reflect differences in virus strain, infection severity, or individual characteristics of the host response remains to be determined. The T cell memory pool remaining after infection is estimated to have a half-life of 8–15 years in humans, and remarkably can be detected after 70 years post small-pox vaccination [51], but the lifespan of influenza-specific T cells has not been tracked past 10 years [52] due to repeated encounters by influenza virus infection making tracking difficult with time.

Multiple doses of LAIVs—and not IIVs or subunit influenza vaccines—can induce long-lived, broad, protective immune responses in mice [53,54,55]. Therefore, LAIV appears to be more immunogenic in animal studies. However, human responses against LAIV are disparate depending on age, and LAIVs only seem more effective than IIV in children, not adults, which coincides with boosted cellular immunity [56,57]. This is possibly a result of children’s more naïve immune states resulting in a robust primary cellular immune response [58,59,60,61,62,63,64,65,66]. LAIV-induced humoral and cellular immunity could be maintained for at least one year [67], but the longevity beyond 1 year has not been studied so far.

The LAIV vaccine currently has limited efficacy, with a reported vaccine efficacy of only 3% for LAIV vs. 65% for IIV reported in 2017 [7]. However, LAIV was a sincere attempt at a T cell-inducing vaccine by nasal delivery, but appears too mild, with reduced tissue tropism and inflammation [68] and has so far failed to increase cellular immunity or improve vaccine efficacy in adults. Therefore, future universal vaccines must improve upon LAIV by either the use of more immunogenic vaccine vectors (such as E1 deleted adenovirus [69] or MVA [70]), adjuvants (interleukin-15 (IL-15) [29], Pam2Cys [71], or MF59 [72]), or less attenuated influenza viruses (such as the use of codon bias mutants [73], NS1 mutants [74], or HA-signal peptide viruses [75]). Lessons should be learned from the limited efficacy and immunogenicity of LAIV in human studies in the development of next-generation universal vaccines.

T cells do not operate in isolation, but in synergy with multiple immune mechanisms to provide broad heterosubtypic protection (Figure 1). Heterologous protection mediated by cross-reactive CD8^+^ T cells was shown to be dependent on an interaction with macrophages and FcR-dependent non-neutralizing antibodies [76], similar to the inexplicable protection rates beyond T cell response magnitude observed in the LAIV Thailand/Philippines clinical trial [77]. In addition, the timing of recruitment of memory CD8^+^ T cells, CD4^+^ T cells, NK cells, or neutralizing antibodies impacted H7N9 infection outcomes [30]. Further cell types also contribute to heterologous protection, such as mucosal associated invariant T (MAIT) cells [78] and FcR^+^ cells to recruit T cell responses [76]. Thus, the next generation of T cell vaccines should combine multiple immune responses for heterologous protection.

## 3. Helping Put the Spotlight on Memory CD4^+^ T Cell Responses

CD4^+^ T cells are phenotypically diverse, and depending on their surface receptors and cytokine expression, can be further characterised into numerous subsets: Th1, Th2, Treg, T follicular helper (Tfh), Th17, Th22, and Th9 (reviewed in [79]). The CD4^+^ T cell field is less developed than the CD8^+^ T cell field (reviewed in [80]) for influenza research, due to the availability of MHCI tetramers and transgenic mouse strains. However, continued efforts using adapted methods such as peptide scanning or whole virus for identification of influenza-specific CD4^+^ T cells have revealed their intrinsic necessity for heterologous protection against influenza virus infection [49]. Characteristically, Th1 type CD4^+^ T cells can secrete IFN-γ and are necessary for the formation of CD8^+^ T cell memory capable of recall and Tfh CD4^+^ T cells for the induction of high affinity class switched B cell memory [81,82]. Induction of HA-stem-specific antibodies requires highly developed B-cell receptor sequences that have undergone affinity maturation, likely to be coordinated by CD4^+^ T follicular helper (Tfh) cells [83]. Immunization with current IIVs activates Tfh cells, correlating with an increase in the number of peripheral antibody-secreting and memory B cells [84]. An emerging body of work has demonstrated the critical role Tfh CD4^+^ cells play in shaping the antibody profile, whereby conserved nucleoprotein (NP)-specific CD4^+^ Tfh cells compete with HA-specific Tfh cells, resulting in reduced H7-HA antibody recall responses after priming with the H1-HA [85,86,87]. Therefore, the order of priming for establishing immunodominance hierarchies for CD4^+^ Tfh responses can impact the development of HA-stem antibodies in the germinal centre. The interplay of protein-specificity and order of immune memory or “imprinting” is important in the context of human immune memory, which is heterogeneous and individualistic depending on a subject’s exposure background.

Imprinting of HA-subtype specific CD4^+^ T cell and B cell memory can have an epidemiological impact. HA-imprinting was recently described in an epidemiological model explaining the age association of H7N9 and H5N1 infections, and providing >75% protection from severe infection caused by these viruses. This was attributable to an individual’s first HA-group encounter resulting in an age distribution of cases born after 1968 for H5N1 and before 1968 for H7N9 corresponding to the switch from a phylogenetic group 1 (including H1, H2, and H5 HA subtypes) to group 2 (includes H3 and H7 HA subtypes) HA during the 1968 Hong Kong H3N2 pandemic [88].

Furthermore, in addition to helper functions, influenza-specific CD4^+^ T cells may be imperative to protection from heterologous influenza infection. In a transgenic mouse model, CD4^+^ T cells have also been found to be directly cytotoxic during influenza infection [89], and human influenza-specific CD4^+^ T cells can express granzymes, perforin, IFN-γ, and exhibit killing function correlating with reduced severity of infection and viral loads [49]. Furthermore, a T cell-inducing vaccine mouse model identified that depletion of memory CD4^+^ T cells but not the CD8^+^ T cell compartment removed heterologous protection from lethal influenza challenges [29]. New roles continue to emerge of the key role influenza-specific CD4^+^ T cells play during heterologous infection.

Overall, influenza-specific CD4^+^ T cells are a cornerstone of effective influenza responses, and their role is a dynamic interplay on the recall of heterologous T and B cell responses and infection outcomes (Figure 1). The long-term role of imprinting CD4^+^ T cell memory responses by universal vaccines will have an important implication in future vaccine design, and is not yet fully explored.

## 4. T Cell-Mediated Protection in Human Studies

Epidemiological studies during past influenza pandemics, where there is an absence of neutralizing antibodies, have suggested that T cell-mediated immunity provided some heterosubtypic protection [90,91,92]. However, the complexity involved in conducting such studies (i.e., obtaining the right cohort that does not have pre-existing antibody response and sampling prior to infection) means that direct evidence for the role of T cells during influenza virus infections in humans has been limited. As such, a major challenge in the universal vaccine field is to determine the strength of a T cell-mediated “correlate of protection”. Unlike animal models, influenza virus infections in humans can result in a wide spectrum of symptoms that reflect either upper or lower respiratory tract involvement. The clinical disease and epidemiology is also distinct between seasonal and avian influenza cases. Thus, in practise, the definition of “protection” in human studies has varied from study to study, and has generally meant a reduction in virus shedding or manifested symptoms. These symptoms have either been self-reported [93,94,95], observed [49], or clinically determined [96,97,98].

In seasonal influenza, the earliest direct evidence that CD8^+^ T cells can mediate protection by facilitating virus clearance after infection was shown in a human challenge study [99]. Thirty years later, Sridhar et al. [94] took advantage of an existing community-wide cohort to show this within the context of natural infections during the H1N1 2009 pandemic. They demonstrated that the magnitude of a subset of IFN-γ^+^IL-2^−^ CD8^+^ T cells was most strongly correlated with reduced disease severity after infection. These cells were also shown to be late-effector memory T cells (CD45RA^+^ CCR7^−^). This was in contrast with the findings from an earlier experimental H3N2 or H1N1 human challenge study, where CD4^+^ T cells were correlated with reduced viral load and disease symptoms instead of CD8^+^ T cell memory responses [49]. This discrepancy was attributed to differences in experimental designs (i.e., virus challenge using laboratory-grown viruses and hemagglutination inhibition (HAI) negative subjects versus natural pandemic infection), and it serves to further highlight the challenges associated with studying T cell responses in humans.

Because T cells recognize highly conserved viral proteins, there has been great interest in investigating their protective role during human cases of avian influenza virus infections. While numerous studies have demonstrated the presence of cross-reactive T cells against avian influenza viruses in vitro [17,18,19,20,86], actual evidence of protection against infection or disease in the field is still lacking since the sporadic and virulent nature of avian influenza cases precludes any systematic cohort studies. Studies to date have been observational, and were based on a relatively small number of cases [30,100] during the H7N9 outbreak in China in 2013. These studies suggested that the early induction and recruitment of memory CD8^+^ T cells was associated with improved prognosis [30,100].

In contrast to cellular immunity data (which are scarce), there have been numerous studies investigating cytokine profiles in infected humans as prognostic markers and signatures of correlates of protection. Pro-inflammatory cytokines such as interleukin (IL)-6 and chemotactic regulators of innate immune cells such as IL-8, MCP-3, and MCP-1 are commonly induced during the acute phase of disease [93,101,102]. However, cytokines that are important in inducing Th1 and Th2 responses have also been reported to be depressed in severely ill individuals [97,98,100]. Of particular interest is IL-12, a regulator of downstream Th1-responses. Concentrations of this cytokine were lower in severe seasonal and avian influenza cases, which incidentally also had lower levels of peripheral CD8^+^ T cells [97,100] compared to mild influenza cases. However, the role of this cytokine needs to be further validated, since data from murine models suggests that high levels of IL-12 cytokine can suppress the formation of lung CD8^+^ memory T cells in the airways [103].

The state of current knowledge of influenza T cell responses in humans is still trying to catch up with the lessons we have learned from animal models. In vitro and murine studies have demonstrated that T cells can mediate lung pathology (reviewed in [104]), primarily through hypersecretion of soluble factors such as IFN-γ and TNF-α, causing direct lysis or “bystander damage” to the lung milieu [105]. Although immune-mediated pathology has certainly been suggested as an important component of severe influenza infections in humans [98,106], a definitive causal role for T cells that is uncoupled from innate immunity or viral factors is still lacking. Indeed, cytokines associated with cellular innate immunity appear to be better predictors of influenza disease severity [93,101,102].

Most studies of T cell responses in humans have been conducted within the peripheral compartment due to the ease of sample access. However, peripheral and airway mucosal immunity are not always in concordance for respiratory infections [93,102]. Furthermore, viral replication can persist in the lower respiratory tract without detection in the upper respiratory tract, where nasal swabs are often collected [107,108] for detection of viral RNA. Airway samples are difficult to collect—particularly those from the lower respiratory tract. Nasal swabs contain mucosal inhibitors and have low cellularity, and are inappropriate for cellular immunity studies, while nasal aspirates are rare in human influenza studies. Furthermore, sampling the lower respiratory tracts through bronchoalveolar lavage is often ethically precluded in healthy adults, and therefore such studies of local respiratory tissue T cell immunity are limited to severe infection cases that compares infection outcome rather than protection from infection or disease [93,97].

In the few rare studies where such data are available, the number of influenza-specific CD4^+^ and CD8^+^ T cells, as well as the magnitude of cytokine response, are far greater in the lungs compared to the blood [98,109,110]. Homologous virus challenge in ferrets that received the monovalent A/Viet Nam/1203/2004 (H5N1) vaccine showed that the CD8^+^ T cells in the airways, but not the blood, correlated with early viral clearance [111]. Elegant studies in murine models have further identified a T cell population in the airways (tissue resident memory T cells, see section below) that are phenotypically and functionally distinct from those in the blood [112] and that have also been identified in humans [113,114]. However, as it is likely that the peripheral compartment will continue to be a proxy of evaluating T cell responses in humans, the extent of how accurately the peripheral T cell responses reflect the airways, and crucially, how they translate to short- and long-term protection are important considerations for effective T cell-mediated vaccine development.

Because most adults have some baseline level of detectable influenza-specific T cells, some efforts have been made to quantitatively identify the T cell-mediated protective threshold as the “correlate of protection”. In a study involving over 2000 children in Thailand and the Philippines, Forrest et al. reported that after receiving two-doses of LAIV, HAI-seronegative children that had ≥100 spot-forming unit (SFU)/10^6^ PBMC in an IFN-γ-ELISPOT assay (using inactivated vaccine components as antigens) were protected against symptomatic disease during subsequent infection [77]. This study also made two notable observations: (i) the protective thresholds for the study populations in these two countries were different, and (ii) other mechanisms of protection not attributed to the measured response were noted with increasing vaccine dosage. Thus, in attempting to provide a “quantitative” correlate of protection, this study also highlighted the population heterogeneity in the T cell compartment and the importance of other immune-mechanisms (i.e., those mediated by non-IFN-γ^+^ cells, or non-HAI-reactive antibodies) that were missed by the experimental approach. In a more recent study, Hayward et al. [95] chose to focus on the immunodominant response against the influenza NP-, rather than the HA-specific T cell response, and reported that ≥20 SFU/10^6^ PBMC was associated with reduced viral shedding in community-acquired influenza. Finally, Sridhar et al. [94] used statistical modelling to predict that every 10-fold increase in the IFN-γ^+^IL-2^−^ CD8^+^ T cell frequency (enumerated by ELISPOT) is associated with a 7-fold reduction in the risk of developing febrile influenza. Because of the different assays used across these three studies, it is difficult to interpret the importance of these quantifiable findings until they are further corroborated. It is worth noting that these studies evaluated symptomatic cases, and evidence for the role of T cells in asymptomatic cases—particularly within the community setting—is still lacking.

Clinical evidence thus far supports the role of existing T cells—particularly the CD8^+^ population—in reducing symptom severity. However, the heterogeneity in experimental design (i.e., study definition, endpoints used, and the use of different or unqualified assays to measure T cell responses, and ELISPOT methods are not directly comparable between different studies) seems to suggest the phenomenon of “The Blind Men and the Elephant”, whereby each study is measuring the T cell response as an immune correlate in different ways, sometimes providing disparate conclusions. Integrating the myriad clinical findings to identify a single quantifiable trait of T cells as the “correlate of protection” remains a major challenge. While some of these issues are surmountable with sophisticated study designs and further research, others—such as airway T cell responses—may remain challenging to address in humans. Thus, in addition to understanding the basic biology, the field must also work on developing a robust system that includes standardized and qualified T cell assays for use in human studies.

## 5. Protect Globally—Think Locally! Trm for Influenza Infection

To date, vaccines intended to elicit T cell immunity against viral infection have generated disappointing levels of protection. This has provoked the recent reassessment of variables important for the successful generation of T cell-mediated immunity with the quantity and location of the memory T cell population now considered of critical importance. T cells require contact with their target cell to mediate their cytolytic function; in other words, they act locally. Therefore, any effective T cell-based vaccine targeting the earliest stages of infection would require the deposition of significant numbers of memory T cells locally within the mucosal tissue. Non-lymphoid tissues house a large proportion of the memory T cell pool [115]. Previously, it was thought that these were simply circulating memory T cells trafficking through the tissue as part of their immunological surveillance. However, it is now accepted that the majority of the cells present within the tissue are in fact resident and represent a distinct memory T cell population [116]. These peripherally deposited memory T cells—termed tissue resident memory (Trm)—play a critical role in local immune protection by directly killing pathogen-infected cells [117,118], releasing cytokines that render the surrounding/local environment non-permissive for pathogen replication [119], and promoting the recruitment of other immune cells from the circulation [120].

Both CD4^+^ and CD8^+^ T cell lineages can form Trm, and can be distinguished from circulating memory T cells due to the expression of key surface markers. CD4^+^ Trm upregulate CD69 and CD11a expression [121,122], while CD8^+^ Trm also express CD69 as well as CD103, the α chain of the αEβ7 integrin [116], although CD103-negative CD8^+^ Trm cells have also been detected [123]; thus, alternative markers for Trm are needed. Not all memory T cell subsets are equally protective against influenza virus infection, with only the Trm pool being shown to be absolutely indispensable for providing optimal heterosubtypic immunity (Figure 2). Following secondary encounter with influenza virus, both CD4^+^ [121,122] and CD8^+^ [124] lung Trm rapidly acquire effector function and respond swiftly, mediating enhanced viral clearance and survival to lethal influenza infection. Why do Trm provide superior protection against influenza virus infection? Simply, they are in the right place at the right time! Influenza virus gains entry into the body by inhalation and initiates its replication cycle within the respiratory tract. The early stages of infection, when virus titres are low in the infected host, provide the ideal window of opportunity for effective immune responses to limit disease progression. Trm deposited along the respiratory tract are perfectly situated to combat the earliest stages of an influenza infection.

Human lung harbours a large number of memory T cells [114], a significant proportion of which express the molecular signature and phenotypic profile of Trm cells. The vast majority of influenza-specific memory CD4^+^ and CD8^+^ T cells present within human lung tissue adopt a Trm phenotype [114,122,125,126,127,128]. Differentiation of these influenza-specific cells into Trm is important for their long-term maintenance, as we find that the size of the influenza-specific CD8^+^ T cell population persisting within the lung directly correlates with the efficiency with which these cells differentiate into Trm [128]. Influenza virus-specific Trm were shown to be highly proliferative and polyfunctional [123,128,129]. Molecular profiling revealed that human lung Trm constitutively express high transcript levels of numerous cytotoxic mediators and deployment-ready mRNAs encoding effector molecules, which is reflective of these cells being poised for rapid responsiveness [123,129]. Influenza virus-specific CD8^+^ Trm in human lung tissue also maintain diverse TCR profiles—a feature important for effective T cell function and protection against the generation of viral-escape mutants [128].

Influenza-specific pulmonary Trm are a core component of the natural heterosubtypic immunity developed following exposure to influenza virus (Figure 2). Thus, influenza vaccines designed to impart optimal heterosubtypic immunity should evoke this memory T cell subset. Defining parameters that promote Trm formation along the respiratory tract is a critical step towards the development of such a vaccine. So, what do we currently know about the factors that drive Trm? Exposure to interleukin-15 (IL-15) [130] and transforming growth factor–β (TGFβ) [131,132], as well as down-regulation of Krüppel-like factor 2 (KLF2), S1P1R [133], T-bet and Eomes expression [130], and up-regulation of Hobit [134] have been proposed as universal Trm developmental requirements. In some tissues (including the lung [125,135,136] and brain [137]), Trm development is also dependent on local cognate antigen recognition.

Can we rationally design vaccines to specifically evoke Trm? In 2012, Iwasaki and colleagues [138] published the first vaccination regime that specifically evoked Trm at a site of potential viral exposure—they coined this vaccination regime “prime-pull”. This approach relied on two steps: conventional parenteral vaccination to elicit systemic T cell responses (prime), followed by the topical application of inflammatory agents to lure (pull) the cells into the tissue where they could differentiate into Trm [138]. While the “prime-pull” vaccination strategy has proven effective at lodging highly-protective Trm pools in the skin [118] and reproductive tract [138], this approach is not effective at depositing Trm cells within the lung [131]. This is because Trm differentiation in the lung—unlike skin and reproductive tract—requires local cognate antigen recognition [125,135,136]. As such, an extension of the “prime-pull” vaccination regime which incorporates the local delivery of cognate antigen was developed as a pulmonary Trm vaccination strategy. Several groups using a variety of modified “prime-pull” vaccination approaches, including (i) intranasal immunization with influenza peptide/protein alone [135,139,140,141] or loaded onto dendritic cells [142], (ii) intranasal delivery of non-replicative influenza virus [143], or (iii) intranasal immunization with monoclonal antibodies linked to influenza antigens that target antigen to respiratory dendritic cells [131], were all able to successfully generate lung CD8^+^ Trm that were highly protective against influenza virus challenge. Collectively, these vaccination studies demonstrate that the key to inducing pulmonary Trm is the local (intranasal) administration of the vaccine antigen (Figure 2). Such strategies might also be beneficial for the optimization of anti-influenza antibody responses through induction of mucosal secretory IgA in the respiratory tract.

Vaccination strategies that deposit influenza virus-specific Trm cells in the lung provide exquisite protection against heterosubtypic influenza challenge [131,143]. Unfortunately, this protection is transient. Unlike populations in the skin and intestinal mucosa, Trm cells in the lung undergo attrition [124] as a result of increased apoptosis [144]. Mouse models confirm that within 7 months of their deposition, influenza virus-specific lung Trm decay below a numerical threshold, leaving the lung susceptible to reinfection [124]. This observed decay of lung Trm cells in mice is no reason to completely dismiss their potential to provide long-term immunity in humans, as just because mouse lung Trm decay does not necessarily mean that human lung Trm also undergoes this rapid rate of attrition. Recent studies by Ahmed and colleagues [145], which utilize in vivo deuterium labelling to assess human memory CD8^+^ T cell turnover and longevity, show that the longevity of circulating memory T cells subsets in humans does not reflect the lifespan of these cells in mice. It will be important to determine the lifespan and turnover rate of human lung Trm if these cells are to be incorporated into vaccines against respiratory pathogens, provide durable vaccine memory, and determine the vaccine schedule to maintain protection. Nonetheless, while lung Trm erode, their counterparts in the upper respiratory tract form a stable long-lived memory T cell pool [142]. Influenza-specific nasal tissue Trm are effective at limiting local replication of influenza virus and can block the transmission of influenza virus from the upper respiratory tract to the lung, and in doing so, prevent the development of severe pulmonary disease [142]. These cells have the potential to provide long-term immunity against influenza virus.

To protect globally against influenza virus, we need to think locally! Trm located along the respiratory tract are perfectly situated to mediate rapid protection following the inhalation of influenza virus, and are capable of providing potent protection against this inhaled pathogen. Influenza vaccines designed to impart heterosubtypic immunity should evoke this memory T cell subset. How best to induce influenza virus-specific Trm along the respiratory tract after vaccination will be one of the challenges to address in the coming years.

## 6. Universal Coverage: Epitopes, HLA, and Ethnicity

The major antigenic targets of influenza-specific T cells are epitopes derived from highly conserved internal virus proteins, providing the basis for heterologous or “universal” immunity across multiple unrelated strains of influenza. Studies assessing the relative contributions of different influenza virus proteins to human T cell responses have identified NP, matrix protein 1 (M1), and PB1 as the major targets [16,49,95], suggesting that a T cell vaccine may only need to focus on a few key viral proteins to achieve similar coverage to natural infection. The focus on priming T cell memory to conserved protein targets may also have implications for reducing interference with HA-imprinting in future vaccine design [88].

T cell responses to influenza virus differ between individuals, primarily due to the expression of diverse MHC alleles (known in humans as human leucocyte antigens, HLAs) that determine the array of viral peptides presented to T cells for recognition. Given the polymorphic nature of HLA alleles [146] and the fact that each individual expresses six HLA Class I (HLA-I; HLA-A, -B, and -C) and six HLA Class II (HLA-II; HLA-DR, -DQ, and -DP) alleles, there is potential for vast diversity in the epitopes presented for recognition during infection. Despite this, T cell responses usually focus only on a few peptide + HLA epitopes, with responses to the same epitopes typically observed across individuals expressing the same HLA allele. Responses to different epitopes often arrange into reproducible immunodominance hierarchies, wherein they can be defined as immunodominant (large) or subdominant (small) in magnitude. The relative size of a given epitope-specific T cell response reflects a complex interplay of virus and host factors (reviewed in [47]), including the particular combination of HLA alleles expressed [147], and does not necessarily relate to the number of naïve epitope-specific precursors available. Approaches combining in vitro infection of cell lines with mass spectrometry analysis have allowed direct identification of the array of peptides presented at the cell surface by individual HLA alleles following influenza infection. To date, these results show the presentation of a reasonably small selection of virus peptides (nine for HLA-A*02:01 and up to six for B*07:02), mainly derived from internal virus proteins, of which only a few (one and two peptides, respectively) consistently induce immune responses [148,149]. Understanding why some presented peptides are targeted while others are seemingly ignored remains a key question that may lead to strategies for optimizing the breadth of vaccine-induced T cell responses.

HLA-I allele expression is an important predictor of cross-reactive influenza-specific CD8^+^ T cell immunity, with a recent study identifying five alleles (A*02:01, A*03:01, B*57:01, B*18:01, and B*08:01) capable of eliciting robust CD8^+^ T cell responses against immunogenic NP and M1 peptides that are conserved across all human influenza A virus, including the novel avian-derived H7N9 virus [18]. Strong representation of these “protective” HLA alleles in a population is therefore predictive of universal memory T cell responses with the potential to protect against multiple circulating and novel influenza A virus strains. As HLA profiles are heritable and strongly influenced by ethnicity, the extent of this universal immunity shows an expected ethnic bias, with higher coverage anticipated in Caucasian populations with enrichment of these key alleles (57%, based on HLA allele expression) as compared to Indigenous Alaskans and Indigenous Australians (16%) [18,150].

Whilst certain HLA alleles may confer a protective advantage through universal immunity to multiple influenza strains, the expression of other alleles may increase susceptibility to severe influenza disease. The H7N9 study mentioned above also identified four HLA-I alleles (A*01:01, A*24:02, A*68:01, and B*15:01) that target unique H7N9 NP and M1 peptide variants that are unlikely to elicit cross-protective immunity between seasonal and H7N9 infection [18]. Thus, upon infection with H7N9, individuals with these HLA alleles will need time to activate and amplify new primary CD8^+^ T cell responses to distinct H7N9 peptide variants rather than recalling T cell responses generated against seasonal influenza viruses, potentially resulting in longer time to recovery and greater risk of severe disease compared to individuals with pre-existing cross-protective CD8^+^ T cell memory.

Hertz et al. showed that HLA-A*24 alleles also have low binding preference for peptides from conserved regions of the 2009 pandemic H1N1 (pH1N1) virus, and carriage of these alleles correlated with low influenza-specific T cell responses in pH1N1-infected patients [151]. Moreover, at a population level, carriage of HLA-A*24 alleles or the HLA-A*68:01 allele was associated with increased mortality to pH1N1. These “risk” HLA alleles may serve as useful markers to identify individuals or populations that are more likely to have low magnitude and less cross-reactive memory T cell responses, placing them at greater risk of severe influenza disease. Interestingly, HLA-A*24 and A*68:01 alleles are highly prevalent in certain Indigenous populations [152,153], which could explain why these groups experience a higher burden of influenza disease and mortality to seasonal and pandemic influenza [151]. However, risk HLA alleles may also have indirect effects on susceptibility to influenza through associations with other autoimmune or metabolic disorders. For example, HLA-A*24 is associated with diabetes [154,155], a disease with increased incidence in Indigenous populations [156]. Nevertheless, the notion of protective and/or risk HLA alleles has been demonstrated previously for human immunodeficiency virus (HIV) and hepatitis C virus (HCV) [157,158,159,160], and is clearly emerging as a factor in influenza infection linked in part to the capacity of certain HLA alleles to present conserved (protective) or variable (risk) influenza peptides.

Ethnicity is a key determinant of risk for severe influenza disease with Indigenous populations worldwide experiencing a disproportionate burden of morbidity and mortality caused by infection with influenza viruses. During the 2009 H1N1 pandemic, higher hospitalization and morbidity rates were observed for Indigenous people in Canada, the United States, New Zealand, and Australia [161,162,163,164,165]. Likewise, Indigenous populations experienced higher mortality rates during the 1918 pandemic (8.5% compared to 2.5%, worldwide [165]). Higher influenza virus infection rates in Indigenous populations may be related to crowded living conditions, increased rates of chronic disease (lung, heart, and metabolic) and co-morbidities that exacerbate the severity of infection. Nevertheless, recovery from influenza depends strongly on the ability to mobilize multiple arms of the immune system—in particular, an early CD8^+^ T cell response [30]. The severity of influenza disease and prolonged hospitalisation periods observed for Indigenous people may therefore reflect a lack of pre-existing protective CD8^+^ T cell immunity that promotes rapid recovery.

As mentioned, HLA-A*24 alleles—a known risk factor for severe pH1N1 disease [151]—are enriched within several global Indigenous populations [152,153], indicating a possible HLA-related deficiency in T cell responsiveness to influenza that may contribute to the vulnerability of these communities. Notably, the HLA profile of Indigenous Australians is relatively restricted and quite distinct from non-Indigenous Australians, with predominant use of HLA-A*34:01, 24:02, 02:01, 11:01, and HLA-B*13:01, 56:01/02, 15:21, 40:01/02 [153]. As such, the dominant CD8^+^ T cell responses in Indigenous Australians are likely to focus on different peptide+HLA epitopes compared to other ethnicities. Since studies have mostly focused on identifying and characterising immunogenic peptides for common and widely expressed HLA alleles such as HLA-A*02:01, immunogenic influenza epitopes are yet to be defined for most (71%) of the HLA-I alleles found in Indigenous Australians. However, analysis of HLA-A*02:01-restricted M1_58–66_-specific CD8^+^ T cells found comparable magnitude, functional quality, and clonal characteristics in Indigenous Australians and non-Indigenous Australians, suggesting that A*02:01^+^ Indigenous Australians (representing 10–15% of this population) have robust cross-protective T cell immunity to any influenza A virus [45,153]. However, accurately determining the extent and quality of CD8^+^ T cell immunity in Indigenous Australians and other Indigenous populations worldwide will require the identification of prominent T cell targets for the relevant Indigenous HLAs. It will be of great interest to see how effectively these HLAs elicit influenza-specific CD8^+^ T cell immunity. Based on HLA profile, targeting responses to a few prominent HLAs in Indigenous Australians could achieve high levels of population coverage [153], but may necessitate a tailored vaccine, as many of these HLA alleles occur rarely in other ethnicities. Clearly, much needs to be done to improve our understanding of influenza virus infection in Indigenous populations before we can design better protocols to protect these populations, which are at greater risk of severe influenza disease and death. Despite the challenges of HLA diversity and possible confounding associations with metabolic and autoimmune disorders, understanding T cell responsiveness to influenza across a broad range of HLA profiles will be an important part of designing and testing the efficacy of future vaccines.

Although response magnitude—typically measured in the periphery—is undoubtedly a critical aspect of effective anti-influenza T cell immunity [49,94], polyfunctionality in the T cell response is also linked with improved outcome to infection [166,167]. HLA genotype may be an important intrinsic factor shaping the magnitude and functional profile of T cell responses. HLA-B alleles are significantly better than HLA-A alleles at generating robust polyfunctional (IFN-γ and IL-2) CD8^+^ T cell responses to HIV, CMV, EBV, and influenza [168]. While this reflects a general enhanced effect for HLA-B alleles, there also appears to be hierarchy of association with polyfunctionality amongst HLA-B alleles. The combination of HLA alleles expressed can also impact the number and TCR repertoire of epitope-specific precursors, while altered peptide presentation, modulation of surface expression, and competition for overlapping peptides can shape the activation and proliferation of these precursors during infection in the context of different HLA profiles [147,169,170,171,172,173,174,175]. It is therefore very difficult to predict T cell response hierarchies to an epitope across the HLA-diverse human population. However, focusing on the largest immunodominant T cell responses may not be all-important, as smaller subdominant T cell epitopes can also contribute to influenza virus clearance, and while they may be underutilized following natural infection, they could be harnessed by vaccination to achieve their full antiviral potential and provide a broad combined response that does better than natural infection [38]. Increasing the breadth of the antiviral response by targeting subdominant epitopes may also reduce the potential for mutational escape in immunodominant epitopes [148,176].

While an effective influenza vaccine should capitalize on the ability of protective HLA alleles to present universally conserved viral components by self-selecting peptides for presentation and elicit broad spectrum T cell immunity, singling out specific peptides as vaccine targets could come at the cost of population-wide coverage across diverse HLA profiles. Approaches that incorporate whole protein antigens are more likely to provide coverage in the human setting of diverse HLA types and avoid concentrated immune pressure that may drive the emergence of escape mutations in targeted epitopes. Furthermore, strategies could be used to optimize peptide presentation by risk HLA alleles through modifications of extra-epitopic processing sites by vaccine design. Upstream extra-epitopic sequences have already been shown to alter CD8^+^ T cell responses to the immunodominant A2-M1_58–66_ CD8^+^ T cell response, possibly via changes to the cleavage pattern of the M1 protein that influence the extent of M1_58–66_ peptide presentation [177].

An ideal influenza T cell-based vaccine would therefore induce an overall robust response comprised of multiple cross-reactive epitope-specific T cell populations with high functional quality and engage multiple immune mechanisms for greater synergy of protection (Figure 2). It would encompass the importance of HLA coverage even for minor ethnicities with rare HLA types, and importantly, leverage our knowledge of key epitopes and cross-reactive T cell responses to do better than nature.

## 7. Universal Vaccine May Still Need to Thwart Viral Escape

Influenza is an RNA virus, and uses its own error-prone RNA-dependent RNA polymerase for replicating its genome. Therefore, the virus is able to adapt rapidly, and is infamous for antigenic drift and resistance to anti-virals under selection pressure. Virus escape from immune-mediated control undermines effective vaccination, and will also need to be considered for a T cell-inducing vaccine. While T cell-targeted proteins and peptides are more highly conserved than the antibody targets in the surface HA, variation and adaptation in T cell epitopes derived from influenza viruses have already been identified. CD8^+^ T cell escape has been observed in some T cell epitopes of naturally circulating influenza viruses [45,177,178,179], which raises the concern for vaccine-mediated T cell escape. As T cells recognize viral peptides within host MHC, viruses can mutate at MHC-anchor residues to reduce presentation [180,181,182,183,184]. Alternatively, exposed residues can form contacts with the TCR for recognition, and variation at TCR contact sites can reduce recognition by existing T cell responses [176,185]. However, due to TCR diversity, establishing a new T cell response to the new peptide variant is often subsequently possible [169,176,182,183,186,187]. Mutation of viral peptides at MHC anchor residues or TCR contacts can impact viral clearance [183] and the recall of established heterosubtypic memory T cell responses [176,180]. However, this concept remains under-appreciated, and could undermine T cell-targeted vaccine-mediated control.

Our previous preliminary data from the isolation of viral RNA from the lungs of infected mice shows that influenza escape can occur very early after the infection of an individual mouse and is common across individual mice, and thus is not a rare event [180]. Mutations at anchor sites and TCR contacts for CD8^+^ T cell influenza epitopes were readily identified, and reverted in the absence of epitope-specific immune pressure [180], which may suggest HLA frequencies in the population will determine the rate of T cell-mediated immune escape. Population sequence studies of drift in the NP protein have identified positive selection pressure [28], and furthermore, at the epitope level, anchor mutations were identified in the HLA-B*27:05- and B*08:01-restricted NP_383–391/380–388_ epitope [179]. Other evidence of immune escape in NP and M1 CD8^+^ T cell epitopes showed that epitopes are functionally constrained, limiting the extent of variation possible, but T cells posed an evolutionary pressure on influenza virus [28,188].

The influenza M1_58–66_ epitope reproducibly selects a dominant public TRAV27/TRBV19^+^ TCRαβ in HLA-A*02:01^+^ donors that can cross-recognize naturally occurring M1_58–66_ peptide variants, seemingly limiting the establishment of mutant strains within the circulating pool of human influenza A viruses and conferring universal immunity to influenza in A*02:01^+^ individuals [45]. Conversely, in the immunodominant HLA-B*07/B*35:01 restricted NP_418–426_ epitope, a different pattern of sequential viral variation has generated over 20 different peptide variants at TCR contact sites [189], with T cell evasion occurring for selected peptides [169,186], resulting in the need for priming multiple T cell sets for the coverage of diverse NP_418_ variants. In this situation, successive waves of exposure to NP_418–426_ variants may drive diverse and unique TCRαβ repertoire recruitment with variable patterns of cross-reactivity against individual variants that favours, rather than controls, the emergence of additional mutants [45,189]. Therefore, pre-emptive priming of T cell subsets to common escape variants could circumvent immune escape [176], and thus could be incorporated into future vaccine strategies that will need to consider contrasting patterns of epitope variation and immune selection. A tailored mosaic peptide vaccine might be the best way to circumvent influenza virus escape, but on a population level for vaccine rollout would be impractical considering HLA selection for full-length proteins and immune competition during T cell priming, this area of research needs further exploration. The H5-derived NP protein is already able to be interchanged in the Leningrad LAIV backbone, demonstrating the possibility of a mosaic approach to incorporating T cell peptide variants with current methods [190].

Furthermore, a recent study demonstrated that HIV-infected children, as opposed to HIV-infected adults, were able to generate HIV variant-specific CD8^+^ T cell responses limiting the selection of escape-epitope variants early in infection [191]. A superior immune response against natural influenza virus infections has also been observed in children aged 4–14, but is not well understood [192], identifying a key window for vaccination of the T cell compartment to do better than nature. Together, these studies suggest that including common TCR escape variant epitopes in a T cell-inducing influenza vaccine might be most effective in young children and could prevent vaccine-mediated T cell escape.

Overall, more research is needed to establish whether combining T cell epitopes, TCR escape variants, and timing of vaccination will benefit T cell-inducing influenza vaccines. In addition, little is known about CD4^+^ epitope variation during influenza infection due to a paucity of defined epitopes. Furthermore, data is also lacking on individual human viral quasi-species generated during infection, with only one example of sequencing of T cell peptides from a longitudinal infected paediatric patient-derived viral RNA [180]. This area of research will need to be expanded to enable a vaccine design that thwarts viral escape.

## 8. Current Developments in Influenza Vaccine Strategies and Future Perspectives towards a Universal Influenza Vaccine

**Figure 3:**
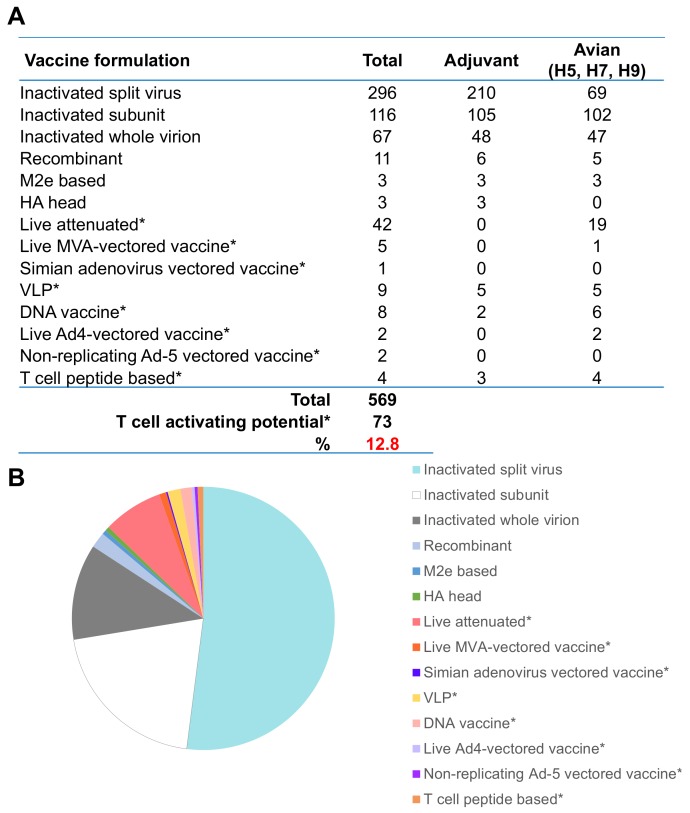
Pre-pandemic vaccines in clinical development. (**A**) Number of vaccines against viruses of pandemic potential in clinical development for human trials. Source from WHO tables on clinical evaluation of Pandemic/potentially pandemic influenza vaccines [193], including those that use adjuvant or are reactive against avian influenza viruses (H5, H7, or H9 subtypes). Vaccines missing input or not defined were excluded. (**B**) Proportion of total vaccines from (**A**). * denotes vaccines which are designed to stimulate T cell response.

While the recent licensure of recombinant HA protein-based vaccines can overcome manufacturing delays and problems with egg-adaptations, they represent only a marginal improvement of an old method based on inducing HA-targeted antibodies. This approach will not improve the breadth of vaccine coverage, and thus new and novel approaches towards influenza vaccination should be considered. T cell immunity has the strongest potential as the immune correlate capable for true universal pan-influenza immunity (Table 1). Current efforts on the clinical development of pandemic vaccines to utilise T cells do not match this potential, and should be further prioritised. Vaccines are currently being developed to utilize T cell-based immune control with the potential for “universal” protection from influenza virus infection. A number of strategies are in clinical development [193] (Figure 3), including the use of vectors such as Modified Vaccinia Ankara (MVA) and Simian Adenovirus encoding the internal NP and M1 proteins of influenza, adenovirus 5 vectored vaccines containing the HA alone, and recombinant peptide approaches for mosaic of conserved peptides or NP with M2e proteins. Viral vectors have different safety profiles to recombinant proteins and inactivated vaccines, but maybe the most promising approach for robust immune responses with the potential to do “better than nature”. Viral vectors can be replicating (such as vaccinia, adenovirus 5, and simian adenovirus) and not safe for use in the immunocompromised and elderly. Non-replicating vectors (including MVA, E1-deleted adenoviruses, influenza minus HA signal peptide) can also be used, and are safe in everyone.

Promising results have been reported for the MVA+NP/M1 vaccine, boosting robust T cell responses in adults and the elderly [70] and protecting from influenza challenge [194]. Furthermore, T cell-inducing vaccine strategies have also been assessed in ferret transmission models, specifically the HA minus signal peptide vaccine, which shows reduced transmission time and peak viral loads from vaccinated ferrets to naïve ferrets [75].

T cell-inducing vaccines for targeting pandemic potential viruses are a significantly under-utilised resource, with only 13% of pandemic potential vaccines in clinical development capable of inducing robust T cell responses (Figure 3A). The majority (83%) of avian H5N1 and H7N9 pandemic potential vaccine candidates are being produced as inactivated vaccines, with the addition of adjuvant to increase immunogenicity and potentiate T cell responses. Adjuvants are used in 76% of non-T cell-targeted vaccines, while only 13% of T cell-targeting vaccines use adjuvant (Figure 3A). The methods of delivery for T cell-inducing vaccines typically do not require exogenous adjuvant because viral vectors are self-adjuvanting, but their use is constrained in some populations, such as the elderly, young children, and pregnant women.

Squalene-oil-in-water-based adjuvants (i.e., MF59 and AS03) have been licensed for use with some inactivated influenza vaccines in Europe and the US, and have been reported to improve CD4^+^ T cell responses after vaccination, presumably through the robust stimulation of innate immune responses, leading to enhanced antigen presentation [195,196,197]. The MF59 IIV [72] can induce IgG isotype switching in a CD4^+^ T cell-independent manner, while still inducing a robust CD8^+^ T cell response. This relationship may be exploited in the context of CD4^+^ T cell imprinting. There is also a suggestion that these adjuvants can improve CD8^+^ T cell responses. The vaccination of ferrets with MF59 or AS03-adjuvanted, inactivated H5N1 vaccines elicited a stronger CD8^+^ T cell response upon virus challenge compared to unadjuvanted vaccines [111]. However, these adjuvants’ effects on the CD8^+^ T cell populations in humans is limited [198]. Care must also be taken when applying inferences from animal data to the human setting, as the majority of animal studies use naïve animals for testing vaccine responses, whereas only the very youngest in the human population are naïve to influenza virus.

In addition to broadly protective T cell vaccines, various vaccination strategies to elicit HA-stem-specific antibodies are currently under development (reviewed in [199]). However, high concentrations of HA-stem-specific antibodies are required to induce sterilizing immunity [200]. Failure to induce high enough HA-stem-specific antibody titres in some individuals combined with the continuous immune pressure on this region in the rest of the population could eventually lead to unforeseen HA-stem escape mutations. A universal influenza vaccine strategy will greatly benefit from an additional layer of long-lasting broadly-reactive immunity in the form of a T cell component to dampen the severity and limit the spread of an influenza virus that managed to escape the HA stem-specific antibody response. Reciprocally, the induction of additional HA-stem-specific antibodies with vaccination could help prevent vaccine-mediated T cell escape, as most natural influenza viruses will be neutralized before they establish an infection, limiting their exposure to CD8^+^ T cell immune pressure. A true universal influenza vaccine would combine the best of both worlds, as a “one-two” punch against influenza viruses.

## 9. Conclusions

This review has highlighted some under-appreciated aspects of influenza-specific T cell immunity for consideration in the design of a universal vaccine of the future (Table 1). This includes some caveats, such as the potential short half-life of T cell resident memory, T cell immune escape, and the impact of antigen presentation timing for immunodominance hierarchies in the context of HLA alleles and population-wide coverage. Further research is needed in the T cell field to identify their protective capacity and the optimal vaccine design for safe delivery, resulting in the longest duration effective memory population. While statistical models have predicted that a T cell-inducing vaccine is likely to be more effective in antibody naïve T cell-primed subjects for H7N9 infection than seasonal H3N2 viruses [201], wider efficacy trials are needed in combination with clinical data. Another challenge faced by T cell-inducing vaccines is that they will need to improve the standard of care provided by the current inactivated vaccines. Inactivated vaccines and vaccine vectors containing influenza viral proteins elicit different immune correlates of protection, and have so far not been compared head-to-head in a large-scale efficacy trial. Unfortunately, this is not in the foreseeable future when the role of T cells mediating protection from infection are still being fundamentally discussed.

The use of T cell-inducing vaccines will require the standardisation of assays to assess T cell memory. The HAI assay is a relatively simple rudimentary measure of virus-specific antibodies which has been in use for over 70 years, and is now well standardised [202]. In contrast, the study of influenza-specific T cell memory responses from human subjects is undoubtedly more technically-demanding and resource heavy compared to serology approaches. There is currently some concerted effort towards standardizing and streamlining experimental approaches for T cell assays in vaccine studies [203], although it will likely take some time before a standard T cell assay is identified. While T cells have a vast potential for breadth of recognition against influenza viruses, the field is in its relative infancy compared to antibody-based vaccines. A quantum leap will be needed in adapting vaccine production, safety and efficacy trials, and standardisation of assays for their assessment on a large efficacy trial scale for their realization.

## Figures and Tables

**Table 1:** Considerations for the design of a universal T cell vaccine of the future.

Vaccine Requirement	Hurdles	Solutions
Induce protective T cell responses	What are the correlates of protection for T cells? - How to measure? - Quantity? - Phenotype? - Longevity?	- Standardized and qualified experimental methods for measuring T cell responses to infection and vaccination. - Simplified commercial assays that can be performed on peripheral T cells.
	Consensus on an acceptable level of protection for T cell vaccines	- Need to consider the context of pandemic preparedness and threat of zoonotic infections. - Whilst immunity induced may not be sterilizing, a vaccine that reduces symptoms, the length and severity of infection, prevents deaths and hospitalizations, and potentially decreases transmission in the community may bring sufficient health and economic benefits to be worthwhile.
Provide universal influenza immunity	Targeting heterologous T cell responses that induce broad protection against diverse influenza virus strains and subtypes.	- Greater knowledge of influenza epitopes recognised by CD4^+^ and CD8^+^ T cells. - Need to define universally conserved virus components that elicit broad spectrum T cell immunity for incorporation in a T cell vaccine.
	Providing population-wide coverage across diverse HLA profiles and ethnicities.	- Approaches that incorporate whole protein antigens or complete virus rather than selected peptides. - Employ strategies to optimize peptide presentation and boost responses to subdominant epitopes (e.g., modification of extra-epitopic processing sites). - Consider tailored vaccine design for ethnicities with unique or ‘risk’ HLA profiles that place them at high risk of severe influenza disease.
	Circumventing virus escape of T cell immunity.	- Spread immune pressure over multiple epitopes through use of full proteins or whole virus. - Pre-emptively prime T cell subsets to common escape variants at a young age. - Combine with other immune mechanisms such as induction of stem antibodies to reduce immune pressure on T cell epitopes.
Establish local immunity at the site of infection	Seeding durable, effective Trm memory populations in the lung and upper respiratory tract.	- Requires local (i.e., intranasal) administration of antigen. - Further work needed to determine the lifespan and stability of human lung and airway Trm, and their potential to provide durable vaccine memory.
Synergize multiple immune mechanisms	Combining long-lasting broadly-reactive T and B cell immunity.	- T cell vaccines should be used in combination with improved antibody vaccine targeting methods and induce multiple responses (e.g., peripheral CD8^+^ T cells, lung-resident CD8^+^ Trm, CD4^+^T_FH_ cells, CD4^+^ T_H1_, memory B cells and cross-reactive stem-specific antibodies) - In a combinatorial vaccine, it will be important to consider the long-term effects of vaccine imprinting on T cell and B cell responses, particularly in the face of ever-evolving influenza viruses.
